# Gender differences among patients with primary ankylosing spondylitis and spondylitis associated with psoriasis and inflammatory bowel disease in an iberoamerican spondyloarthritis cohort: Erratum

**DOI:** 10.1097/MD.0000000000006485

**Published:** 2017-03-24

In the article “Gender differences among patients with primary ankylosing spondylitis and spondylitis associated with psoriasis and inflammatory bowel disease in an iberoamerican spondyloarthritis cohort”,^[1]^ which appeared in Volume 95, Issue 51 of *Medicine*, Dr. Elsa Vieira Sousa's name was originally misspelled.
